# Long Intergenic Noncoding RNA00265 Enhances Cell Viability and Metastasis *via* Targeting miR-485-5p/USP22 Axis in Osteosarcoma

**DOI:** 10.3389/fonc.2022.907472

**Published:** 2022-05-26

**Authors:** Ting Chen, Jinxin Liu, He Zhang, Jiatong Li, Guanning Shang

**Affiliations:** Department of Orthopedics, Shengjing Hospital of China Medical University, Shenyang, China

**Keywords:** Linc00265, osteosarcoma, miR-485-5p, USP22, viability

## Abstract

Osteosarcoma is one of the bone malignancies in children and adolescents. Long noncoding RNAs (lncRNAs) have been demonstrated to participate in osteosarcoma development and progression. Linc00265 has been shown to involve in osteosarcoma oncogenesis; however, the underlying mechanism is largely unclear. In this study, we investigated the function of linc00265 in osteosarcoma cells, including cell viability, migration and invasion. Moreover, we elucidated mechanistically the involvement of linc00265 in osteosarcoma. We found that linc00265 overexpression promoted viability, migration and invasion of osteosarcoma cells. Notably, linc00265 sponged miR-485-5p and increased the expression of USP22, one target of miR-485-5p, in osteosarcoma cells. Strikingly, linc00265 exerted its oncogenic function *via* regulating miR-485-5p and USP22 in osteosarcoma. Taken together, targeting linc00265 is a promising approach for treating osteosarcoma patients.

## Introduction

Osteosarcoma is one of the common bone malignancies, which often happens in children and adolescents (1, 2). The standard therapeutic strategy is surgery in combination with chemotherapy for osteosarcoma patients (3–5). Notwithstanding the treatment and diagnostic approaches have been improved, some osteosarcoma patients still have worse prognosis due to resistance to chemotherapy and distant metastasis (6–8). Hence, more investigations need to determine the mechanism of osteosarcoma carcinogenesis and discover the new promising agents for improving the efficiency of treatment outcome in osteosarcoma patients.

LncRNA is one type of noncoding RNA without protein encoding functions, which has more than 200 nucleotides (9). LncRNAs affect the cellular functions *via* modulating gene expression at multiple regulatory levels, such as transcriptional and post-transcriptional levels (10, 11). LncRNAs have been demonstrated to play a necessary role in a number of types of cancers, including osteosarcoma (12–15). Evidence has revealed that linc00265 is critically involved in carcinogenesis and tumor progression in multiple type cancers (16). For example, studies have shown that the expression of linc00265 was increased in bone marrow and serum of acute myeloid leukemia (AML) patients, which was associated with poor overall survival (16, 17). Linc00265 regulated proliferation, migration and invasion *via* activation of phosphatidylinositol-3-kinase (PI3K)/AKT signaling pathway in AML cell lines (17). Moreover, linc00265 overexpression increased autophagy and attenuated apoptosis of AML cells *via* sponging miR-485-5p and subsequent upregulation of interferon-regulatory factor 2 (IRF2) (16). Similarly, another study also showed that linc00265 expression was elevated in peripheral blood and bone marrow in leukemia patients (18).

Studies have demonstrated that linc00265 downregulation blocked colorectal cancer (CRC) oncogenesis *via* enhancement of zinc finger MIZ-type containing 2 (ZMIZ2) expression due to sponging several miRNAs, including miR-375, miR-30c-2-3p, miR-324-3p and miR-130-3p, leading to upregulation of ubiquitin specific peptidase 7 (USP7) and stabilization of β-catenin (19). Moreover, Ge et al. showed that linc00265 knockdown attenuated the expression of epidermal growth factor receptor (EGFR) in CRC cells, resulting in suppression of proliferation, invasion and induction of apoptosis in CRC cells (20). Furthermore, linc00265 enhanced glycolysis and lactate production *via* binding to miR-216b-5p and elevating the expression of tripartite motif containing 44 (TRIM44) in CRC (21). Clinically, higher expression of linc00265 was correlated with poorer prognosis in CRC patients, indicating that linc00265 might be an independent prognostic marker (21, 22). In lung adenocarcinoma, linc00265 was uncovered to interact with miR-7 and subsequently upregulate fibroblast growth factor 2 (FGF2), contributing to lung cancer tumorigenesis (23). The role of linc00265 in osteosarcoma has not been fully investigated. Thus, we aimed to determine the function and molecular insight of linc00265 in osteosarcoma progression.

Ubiquitin-specific protease 22 (USP22) can act as a deubiquitinating enzyme and exhibit its implication in oncogenesis due to regulation of proliferation, cell cycle, apoptosis, cancer stemness and chemoresistance (24). USP22 is abnormally expressed in several cancer types and facilitates tumor malignant progression. For example, USP22 can stabilize the E2F6 stability and activate Akt pathway in hepatocellular carcinoma (HCC), leading to aggressive progression of HCC (25). USP22 inhibits HER2-mediated breast cancer aggressiveness *via* reducing the unfolded protein response (26). Moreover, USP22 regulates necroptosis in tumor cells *via* governing receptor-interacting protein kinase 3 (RIPK3) stability (27). USP22 expression was increased in osteosarcoma tissues and linked to osteosarcoma progression (28). The detailed mechanism of USP22-mediated osteosarcoma is still elusive.

In the present study, we investigated the expression and biological functions of linc00265 in osteosarcoma cells. Moreover, we further explored the molecular mechanism of linc00265-mediated carcinogenesis in osteosarcoma. Our study demonstrated that linc00265 promoted cell viability, migration and invasion *via* targeting miR-485-5p/USP22 axis in osteosarcoma, suggesting that linc00265 might be a useful target for osteosarcoma therapy.

## Materials and Methods

### Cell Culture

The osteosarcoma cell lines, MG63 and U2OS, were cultured in Dulbecco’s modified eagle medium (DMEM) medium with 10% fetal bovine serum (FBS). SAOS-2 cell line was cultured in McCoy’s 5A modified medium with 10% FBS. The human osteoblast (HOB) cells were cultured in MEM-F12 medium with 10% FBS. The cells were maintained in the presence of 5% CO_2_ at 37°C.

### CCK-8 Cell Viability Assay

The cell counting kit-8 (CCK-8) kit (Beyotime, Shanghai, China) was used to measure viability of osteosarcoma cells after different treatments. Osteosarcoma cells were seeded on 96-cell plates and incubated in medium for 24, 48, and 72 hours, respectively. Then. 10 μl CCK-8 reagent was added and incubated for 3 hours at 37°C. The OD values were measured at 450 nm by a microplate reader (Sunnyvale, CA, USA).

### Colony Formation Assay

The treated osteosarcoma cells were seeded into 6-well plates and maintained with 5% CO_2_ at 37°C in a humidified incubator for 14 days. The culture media was removed and the cells were fixed with 4% paraformaldehyde for half hour after the cells were washed by PBS. Then, 0.1% crystal violet was added to stain the cell colony for 15 minutes. Finally, we counted the number of cell colony.

### Quantitative Real-Time Reverse Transcription-PCR Analysis

The treated osteosarcoma cells were harvested and total RNA was extracted using TRIzol agent. The mRNA was reverse-transcribed by the cDNA Reverse Transcription Kit (Thermo Fishes, USA) following the manufacturer’s instructions. Then, qRT-PCR was conducted using SYBR Green PCR Master Mix Kit as described previously (REF). The primers are: USP22, forward primer, 5’-AGC CAA GGG TGT TGG TCG CG-3’, and reverse primer, 5’-ACT GCC ACC ACG CCC GAA AG-3’. GAPDH, forward primer, 5′- ACC CAG AAG ACT GTG GAT GG -3′; reverse primer, 5′- CAG TGA GCT TCC CGT TCA G- 3′.

### Western Blotting Analysis

The treated osteosarcoma cells were harvested and lysed in a lysis buffer containing protease inhibitor cocktail. The protein concentration was measured by the bicinchoninic acid (BCA) assay. The protein was determined by SDS-PAGE and probed with antibodies against USP22 (SC-390585, Santa Cruz, USA, 1:1000) and tubulin (#T9028, Sigma-Aldrich, St. Louis, MO, USA, 1:5000) as described previously (29). The quantitative results were analyzed by ImageJ software (NIH, USA).

### Transfection Assay

Osteosarcoma cells were seeded into 6-well plates and transfected with different plasmids by Lipofectamine 2000 as described previously (29). USP22-specific shRNA, USP22 lentiviral particles, miR-485-5p mimics, miR-485-5p inhibitors and control vectors were provided by GenePharma Company (Shanghai, China). After cells were transfected for 48 hours, the cells were subjected to analysis for cell viability, migration and invasion, which were described under the result sections.

### Wound Healing Assay

The treated osteosarcoma cells were seeded on 6-well plates for overnight. Then, we created a wound *via* scratch approach by a 100 μl pipette tip after cell confluence reached to higher than 90%. The wound area was photographed at 0 hour and 20 hours using an inverted microscope, respectively. The distance of wound closure was analyzed by ImageJ software.

### Transwell Invasion Assay

The treated osteosarcoma cells were seeded on the upper layer of 24-well inserts (Corning Incorporated, NY, USA) with 200 μl serum-free medium. The membrane of the upper layer was precoated with Matrigel. In addition, 500 μl complete medium with 10% FBS were added in the under layer. After 20 hours incubation, the invaded cells through the membrane were stained by 4 μg/ml Calcein AM. The invaded cells were photographed by a microscope.

### Luciferase Report Assay

The wild-type or mutant binding sequence of miR-485-5p in linc00265 or USP22 3’UTR was sub-cloned into pmirGLO dual-luciferase vector. The dual luciferase report assay system (Promega, Madison, WI, USA) was utilized to measure luciferase activity as described previously (30).

### Statistical Analysis

Data are presented as mean ± SEM. Two-way ANOVA followed by Tukey’s test was conducted for comparison among multiple groups. Student’s *t*-test was used for comparison between two groups. *P* < 0.05 was considered statistically significant.

## Results

### Linc00265 Overexpression Promotes Viability of Osteosarcoma Cells

**Figure 1:**
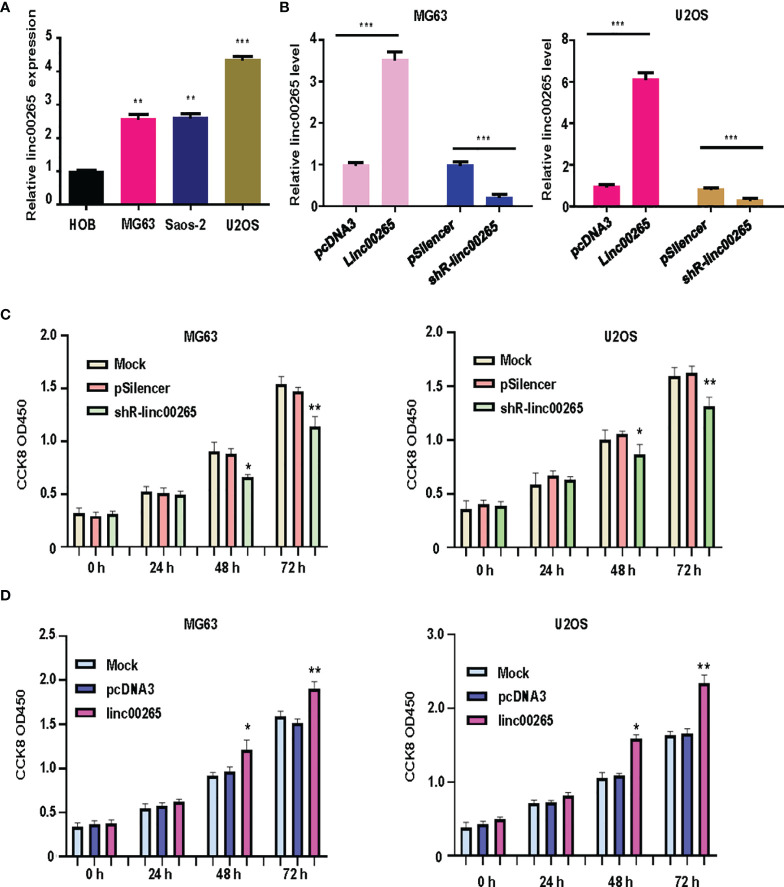
Linc00265 overexpression promotes viability of osteosarcoma cells. **(A)** Real-time RT-PCR was utilized to examine the expression levels of linc00265 in HOB cells and three osteosarcoma cell lines, including MG63, SaOS-2 and U2OS. **(B)** Real-time RT-PCR was performed to examine linc00265 expression levels in MG63 and U2OS cells after various plasmid transfections. **(C)** CCK-8 assay was conducted to detect the viability of osteosarcoma cells after linc00265 knockdown for 24 h, 48 h, and 72 h. **(D)** CCK-8 assay was utilized to detect the cell viability in linc00265-overexpressing osteosarcoma cells for 24 h, 48 h, and 72 h. *P < 0.05, **P < 0.01, ***P < 0.001 vs. control.

**Figure 2:**
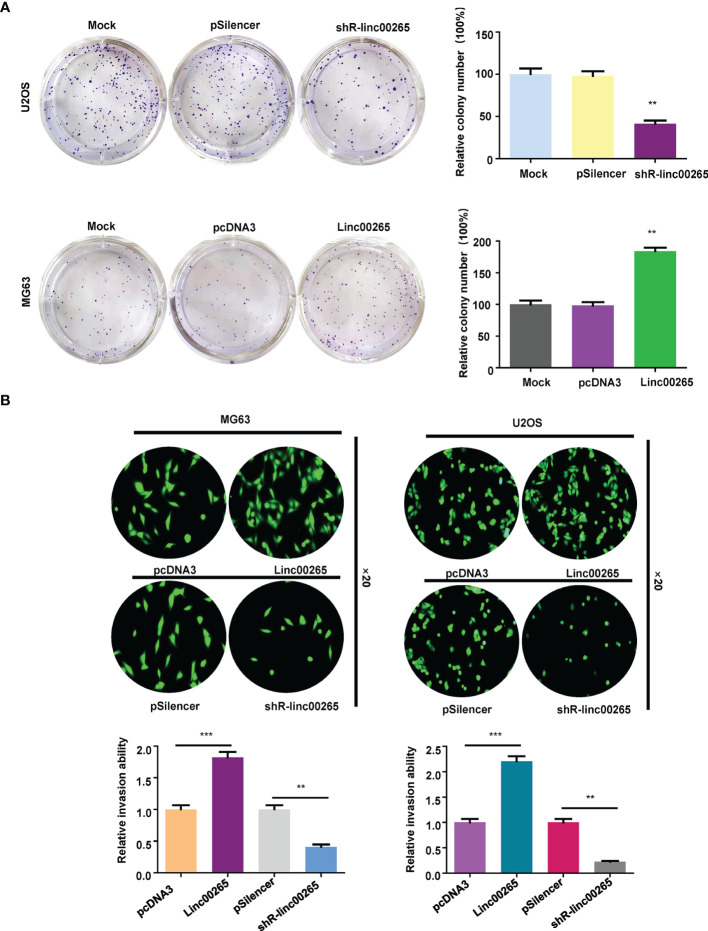
Linc00265 overexpression promotes colony formation and invasion of osteosarcoma cells. **(A)** Colony formation assay was used to detect the ability of colony formation in osteosarcoma cells after linc00265 downregulation (U2OS cells, Top) and upregulation (MG63 cells, Bottom). The quantitative data were illustrated (Right panel). **(B)** Transwell invasion assays were conducted in MG63 and U2OS osteosarcoma cells after linc00265 overexpression or downregulation. The quantitative data of invasion ability were shown (Bottom panel). **P < 0.01, ***P < 0.001 vs. control.

Several studies have uncovered that linc00265 exerts tumor promotive functions in a number of cancer types. We are wondering whether linc00265 upregulation could inhibit viability of osteosarcoma cells. First, we used real-time RT-PCR analysis to detect the expression of linc00265 in normal human osteoblasts (HOB) and osteosarcoma cells (U2OS, MG63 and SW1353). The results showed that linc00265 was highly expressed in osteosarcoma cell lines compared with HOB cells (**Figure 1A**). To investigate the biological function of linc00265 in osteosarcoma cells, we transfected linc00265 plasmid and shR-linc00265 into MG63 and U2OS cells. As shown in **Figure 1B**, MG63 and U2OS cells after transfection with linc00265 plasmid exhibited higher expression of linc00265 compared with pcDNA3 transfection (**Figure 1B**). Osteosarcoma cells treated with shR-linc00265 plasmid displayed the lower expression of linc00265 compared with PSilencer transfection in both MG63 and U2OS cell lines (**Figure 1B**). Next, we aimed to explore the cell viability in MG63 and U2OS cells after linc00265 modulation by CCK-8 assay. The results from CCK-8 assay clearly demonstrated that downregulation of linc00265 attenuated cell viability in U2OS cells and MG63 cells (**Figure 1C**). In line with this finding, upregulation of linc00265 facilitated viability of osteosarcoma cells in both osteosarcoma cell lines (**Figure 1D**). Moreover, colony formation assay was performed to examine the ability of colony formation in both MG63 cells and U2OS cells after linc00265 changes. We observed that downregulation of linc00265 suppressed colony formation capacity in U2OS cells, while overexpression of linc00265 stimulated colony formation ability of MG63 cells (**Figure 2A**). Taken together, linc00265 governs cell viability and colony formation in osteosarcoma cells.

### Linc00265 Overexpression Enhances Invasion and Migration of Osteosarcoma Cells

**Figure 3:**
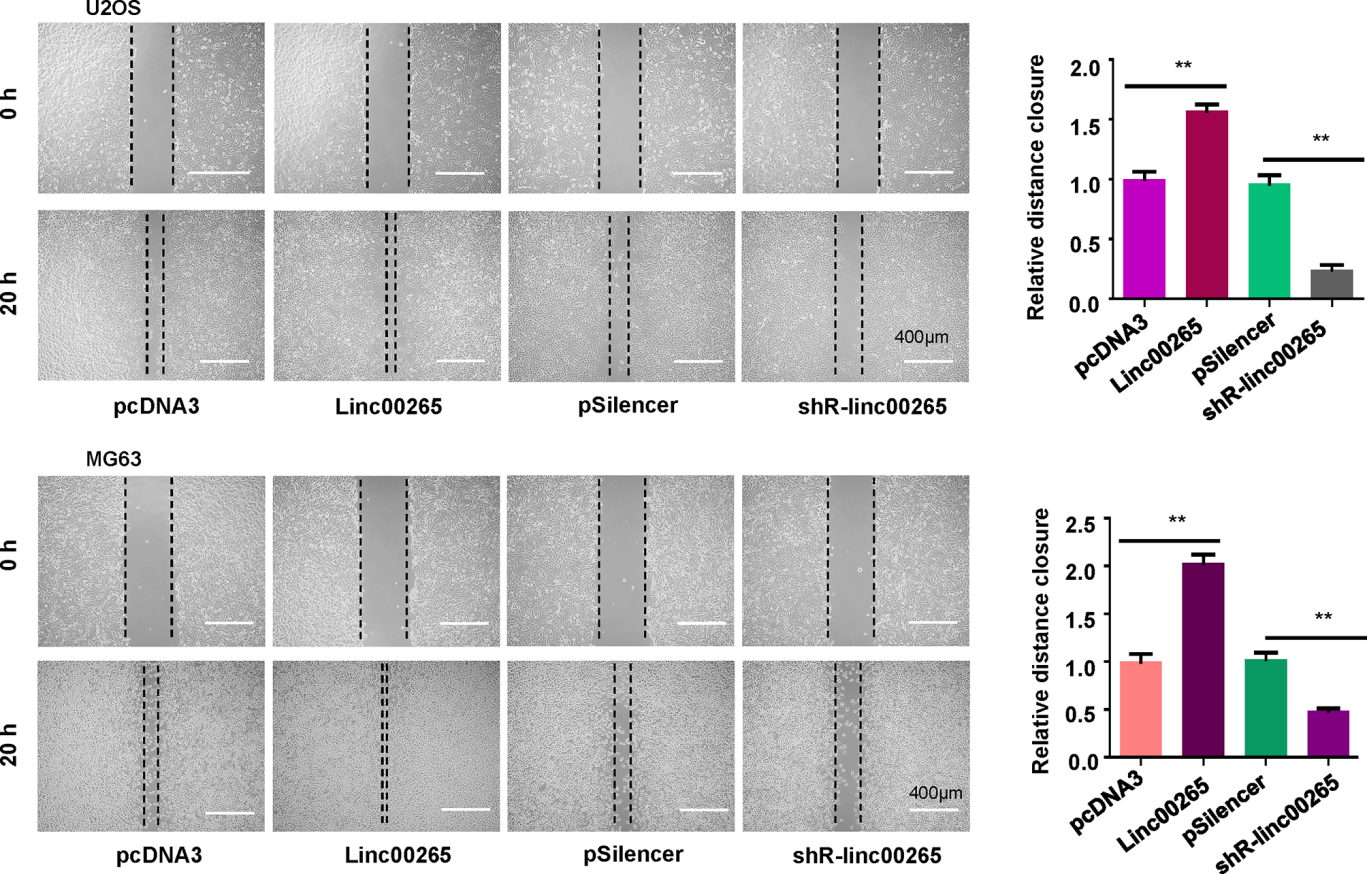
Linc00265 overexpression promotes migration of osteosarcoma cells. Wound healing assay was utilized to examine the cell migratory ability in U2OS and MG63 osteosarcoma cells after linc00265 modification (Left panel). The quantitative data of wound healing assays were demonstrated (Right panel). **P < 0.01 vs. control.

Studies have reported that linc00625 regulates migratory and invasive capacities in a spectrum of cancers. Here, we explored the invasiveness and migration of osteosarcoma cells after linc00625 overexpression and depletion. We observed that linc00265 plasmid transfection facilitated cell invasion in MG63 and U2OS cells (**Figure 2B**). Consistently, shR-linc00265 transfection reduced the cell invaded numbers through Matrigel membrane in both osteosarcoma cell lines (**Figure 2B**). Next, wound healing assays were conducted to examine the motility of osteosarcoma cells after linc00265 upregulation or downregulation. As demonstrated in **Figure 3**, increased linc00265 promoted closure in wound area in osteosarcoma cells. In contrast, decreased linc00265 retarded closure of wound area in MG63 and U2OS cells (**Figure 3**). The wound healing data indicated that linc00265 could control migratory and invasive capacity in osteosarcoma cells.

### Linc00265 Sponges miR-485-5p in Osteosarcoma Cells

**Figure 4:**
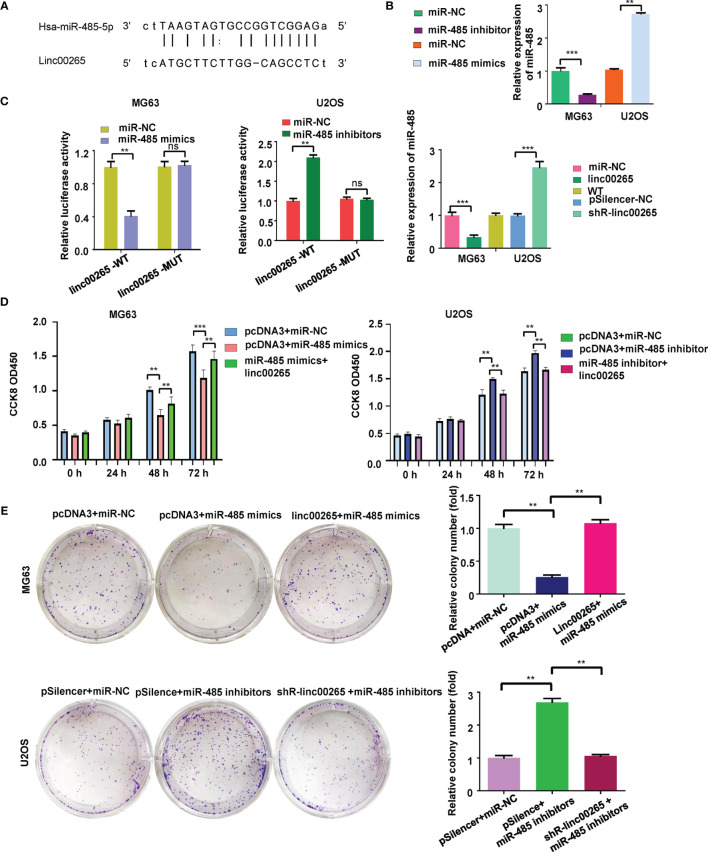
Linc00265 sponges miR-485-5p in osteosarcoma cells. **(A)** The target gene of linc00265 was predicted by starbase database. **(B)** The efficacy of transfection was confirmed by qRT-PCR in MG63 and U2OS osteosarcoma cells. **(C)** Luciferase reporter assay was used to confirm miR-485-5p as a target of linc00265 (Left and middle panels). The expression of miR-485 was detected by qRT-PCR in MG63 and U2OS osteosarcoma cells after linc00265 changes (Right panel). **(D)** Cell viability was examined by CCK-8 assay in MG63 and U2OS osteosarcoma cells after linc00265 modification and miR-485-5p changes. **(E)** The colony formation ability was measured in MG63 and U2OS osteosarcoma cells after linc00265 modification and miR-485-5p changes (Left panel). The quantitative data of colony formation ability were demonstrated for colony formation (Right panel). **P < 0.01, ***P < 0.001 vs. control. ns, no statistic difference..

It has been known that linc00265 often sponges specific miRNAs to regulate its downstream genes. The potential miRNAs that might bind with linc00265 were predicted using starBasev2.0. From this database, we predicted that miR-485-5p could be a sponging miRNA of linc00265 because this lncRNA has a position interacting with miR-485-5p (**Figure 4A**). We transfected miR-485-5p mimics and inhibitors into osteosarcoma cells and observed that miR-485-5p expression was elevated in mimics-treated group, while miR-485-5p levels were downregulated in inhibitor-treated group (**Figure 4B**). To validate whether the miR-485-5p is the downstream target of linc00265, the luciferase reporter assay was performed using a wild-type or mutant target site from linc00265. We found that miR-485-5p mimic decreased luciferase activity in the wild-type linc00265 reporter, but not in mutant linc00265 reporter (**Figure 4C**). At the same time, we uncovered that the expression levels of miR-485-5p were decreased in osteosarcoma cells after linc00265 overexpression (**Figure 4C**). Meanwhile, the levels of miR-485-5p were elevated in osteosarcoma cells after linc00265 depletion. Therefore, linc00265 might target miR-485-5p in osteosarcoma cells.

### MiR-485-5p Mimics Inhibits Cell Viability, Which Is Abrogated by linc00265

To evaluate the association between miR-485-5p and linc00265 in osteosarcoma cells, MG63 and U2OS cells were transfected with miR-485-5p mimic, miR-485-5p inhibitor and matched controls. The data from CCK-8 assay suggested that miR-485-5p mimics repressed viability of osteosarcoma cells (**Figure 4D**). Meanwhile, miR-485-5p inhibitors facilitated cell viability in both MG63 and U2OS cells. Moreover, miR-485-59-mediated effect on cell viability was abrogated by linc00265 overexpression in osteosarcoma cells (**Figure 4D**). To further confirm this phenotype, colony formation assay was utilized in osteosarcoma cells after co-transfection of miR-485-5p mimics and linc00265 plasmid. The results indicated that miR-485-5p mimics attenuated colony formation ability of MG63 cells, which was rescued by linc00265 overexpression (**Figure 4E**). Consistently, miR-485-5p inhibitor treatment enhanced colony formation capacity of U2OS cells, which was abolished by shR-linc00265 infection (**Figure 4E**).

### USP22 Is a Direct Target of miR-485-5p

**Figure 5:**
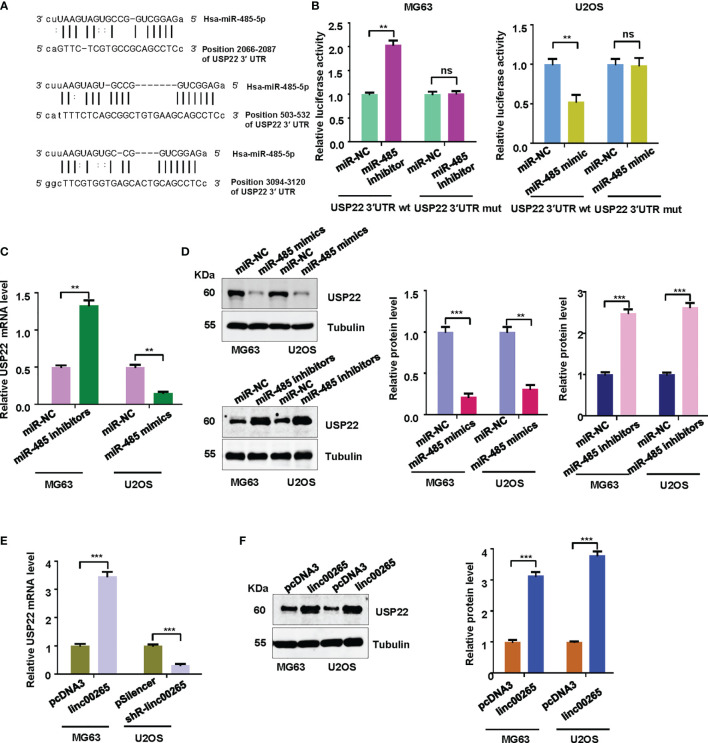
USP22 is a downstream target of miR-485-5p. **(A)** The TargetScan database was utilized to predict the downstream target of miR-485-5p. USP22 3’UTR has the complementary binding sites with miR-485-5p. **(B)** Luciferase reporter assay was used to confirm USP22 as a target of miR-485-5p in MG63 and U2OS cells. **(C)** The expression of USP22 mRNA levels was detected by qRT-PCR in MG63 and U2OS osteosarcoma cells after miR-485 changes. **(D)** The expression of USP22 protein levels was detected by Western blotting in osteosarcoma cells after miR-485 changes (Left panel). The quantitative data were demonstrated for USP22 protein levels (Right panel). **(E)** The expression of USP22 mRNA levels was detected by qRT-PCR in osteosarcoma cells after linc00265 changes. **(F)** The expression of USP22 protein levels was detected by Western blotting in osteosarcoma cells after linc00265 changes (Left panel). The quantitative data were demonstrated for USP22 protein levels (Right panel). **P < 0.01, ***P < 0.001 vs. control. ns, no statistic difference.

Evidence has demonstrated that miRNAs regulate the expression of targets due to that miRNAs contain special sequences that are complementary to downstream targets. The TargetScan database was utilized to predict the downstream targets of miR-485-5p. We found that USP22 3’UTR has the complementary binding sites with miR-485-5p (**Figure 5A**). Luciferase reporter results revealed that miR-485-5p mimic decreased luciferase activity for the WT USP22 reporter, but not for mutant USP22 reporter. Meanwhile, miR-485-5p inhibitor increased luciferase activity for the WT USP22 reporter (**Figure 5B**). RT-PCR was performed to examine the expression of USP22 mRNA in osteosarcoma cells after miR-485-5p mimic treatment or inhibitor exposure. As demonstrated in **Figure 5C**, miR-485-5p mimic treatment reduced the USP22 mRNA expression in U2OS cells, while miR-485-5p inhibitor enhanced the USP22 mRNA levels in MG63 cells. Western blotting data further validated that miR-485-5p overexpression reduced the expression of USP22 protein, whereas miR-485-5p downregulation increased the USP22 protein levels in osteosarcoma cells (**Figure 5D**). To check whether linc00265 could regulate the expression of USP22, RT-PCR and western blotting analysis were utilized to test the expression of USP22 in osteosarcoma cells after linc00265 changes. Our results suggested that linc00265 upregulation increased the expression of USP22 at mRNA and protein levels, and linc00265 downregulation attenuated the USP22 expression levels in osteosarcoma cells (**Figures 5E, F**).

### Linc00265 Exerts Its Functions *via* miR-485-5p/USP22 Axis

**Figure 6:**
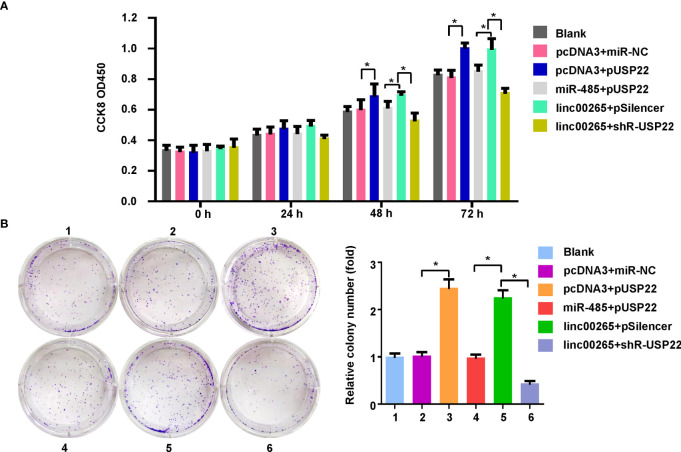
Linc00265 promoted cell viability and colony formation *via* miR-485-5p/USP22 axis. **(A)** Cell viability was examined by CCK-8 assay in osteosarcoma cells after linc00265 modification, miR-485-5p changes, or USP22 modification or combination. **(B)** The colony formation ability was measured in osteosarcoma cells after linc00265 modification, miR-485-5p changes, or USP22 modification or combination. The quantitative data were demonstrated for colony formation (Right panel). 1: Blank. 2: pcDNA3 + miR-NC. 3: pcDNA3 + pUSP22. 4: miR-485 + pUSP22. 5. Linc00265 + pSilencer. 6. Linc00265 + shR-USP22. *P < 0.05 vs. control.

**Figure 7:**
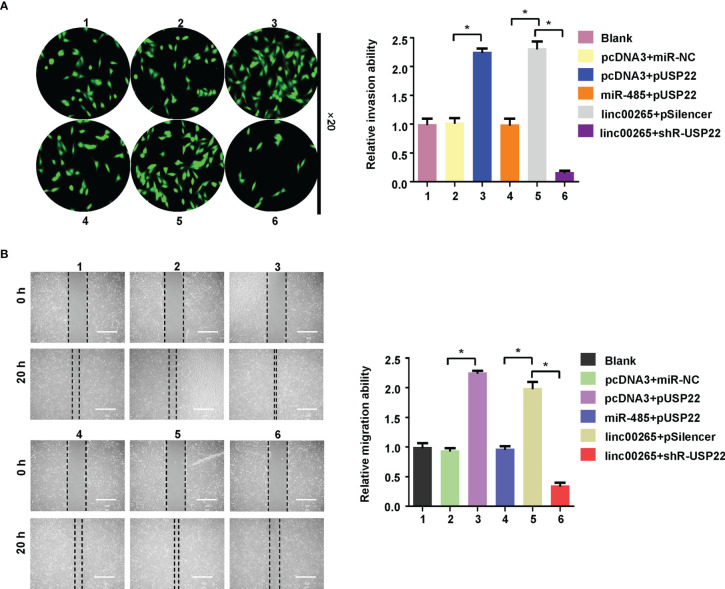
Linc00265 enhanced migration and invasion *via* miR-485-5p/USP22 axis. **(A)** Cell invasion was examined by Transwell invasion assay in osteosarcoma cells after linc00265 modification, miR-485-5p changes, or USP22 modification or combination. **(B)** Cell migration was measured by wound healing assay in osteosarcoma cells after linc00265 modification, miR-485-5p changes, or USP22 modification or combination (Left panel). The quantitative data were demonstrated for wound healing assay (Right panel). 1: Blank. 2: pcDNA3 + miR-NC. 3: pcDNA3 + pUSP22. 4: miR-485 + pUSP22. 5. Linc00265 + pSilencer. 6. Linc00265 + shR-USP22. *P < 0.05 vs. control.

Lastly, we examined whether miR-485 and USP22 were involved in linc00265-mediated oncogenic function in osteosarcoma cells. MG63 cells were transfected with miR-485-5p or USP22 plasmid or linc00265 plasmid or shR-USP22 or combination treatments. Then, CCK-8 assay, colony formation, wound healing assay, and Transwell invasion assays were done to determine cell viability, colony formation ability, migratory capacity and invasive ability in osteosarcoma cells, respectively. CCK-8 data showed that USP22 overexpression accelerated viability of osteosarcoma cells, which was rescued by miR-485-5p upregulation (**Figure 6A**). Overexpression of linc00265 increased cell viability in MG63 cells, which was abolished by downregulation of USP22 (**Figure 6A**). In line with this result, USP22-mediated promotion of colony formation was abrogated by overexpression of miR-485-5p. Moreover, linc00265-induced colony formation was rescued by inhibition of USP22 in osteosarcoma cells (**Figure 6B**). Furthermore, USP22-induced cell migration and invasion were abolished by miR-485-5p overexpression in osteosarcoma cells (**Figures 7A, B**). Linc00265-triggered motility of osteosarcoma cells was blocked by upregulation of miR-485-5p in osteosarcoma cells (**Figures 7A, B**). Altogether, linc00265 exerted its functions in part *via* regulation of miR-485-5p/USP22 axis.

## Discussion

A line of evidence uncovered that linc00265 plays an essential role in tumorigenesis. Yang et al. revealed that linc00265 stimulated cell proliferation *via* interaction with miR-144-3p and increasing chromobox 4 (CBX4) in gastric cancer (31). In bladder cancer cells, linc00265 was found to facilitate cell viability, proliferation and migratory ability *via* inhibition of miR-4677-3p and promotion of fibroblast growth factor 6 (FGF6) expression (32). Xiao and colleagues reported that linc00265 facilitated cell angiogenesis *via* sponging miR-382-5p, leading to upregulation of Spermidine/spermine acetyltransferase-1 (SAT1) and vavguanine nucleotide exchange factor 3 (VAV3) in osteosarcoma, which caused suppression of proliferation, motility and angiogenesis in osteosarcoma cells (33). Moreover, linc00265 was highly expressed in osteosarcoma patients and correlated with a poor prognosis (33). Here, we reported that linc00265 facilitated osteosarcoma progression *via* targeting miR-485-5p/USP22 axis.

Evidence has shown that miRNAs critically participate in oncogenesis of numerous types of cancer, including osteosarcoma. For example, miR-485-5p retarded cell proliferation and metastasis *via* inhibition of CX3CL1 in osteosarcoma cells (34). Similarly, one study reported that miR-495-5p targeted heat shock protein (Hsp90) expression and inactivated Akt1 phosphorylation and blocked PI3K/Akt pathway, resulting in suppression of cell proliferation in osteosarcoma (35). Another study showed that miR-485-5p can downregulate the expression of baculoviral IAP repeat containing 5 (BIRC5) and block the malignant phenotype of osteosarcoma (36). In our study, we found that miR-485-5p inhibited the expression of USP22 in osteosarcoma cells, leading to suppression of viability and motility of osteosarcoma cells.

USP22 promoted tumor development and progression in certain cancer types. For instance, USP22 enhanced cell proliferation *via* increasing surviving stability in renal cell carcinoma (RCC) cells (37). In pancreatic ductal adenocarcinoma (PDAC) cells, USP22 stimulated cell growth *via* targeting dual-specificity tyrosine regulated kinase 1A (DYRK1A) (38). In breast cancer cells, USP22 positively regulated ERα expression *via* maintaining its stability (39). Moreover, USP22 downregulation repressed cell proliferation, invasion and epithelial-mesenchymal transition (EMT) *via* inactivation of PI3K/Akt signaling pathway in osteosarcoma cells (28). Moreover, inhibition of USP22 reduced osteosarcoma tumor growth and metastasis in mice (28). Liu et al. also found that miR-140 attenuated osteosarcoma progression *via* interference of USP22-involved lysine-specific demethylase 1 (LSD1) stabilization and elevating p21 expression (40). We also confirmed that USP22 enhanced viability and motility of osteosarcoma cells.

## Conclusions

In summary, linc00265 promoted cell viability, migration and invasion in osteosarcoma cells, indicating that linc00265 is an oncogene in osteosarcoma. Moreover, linc00265 sponged miR-485-5p and suppressed its expression in osteosarcoma cells. Furthermore, we identified that USP22 is a direct target of miR-485-5p in osteosarcoma cells. Thus, miR-485-5p/USP22 axis was critically involved in linc00265-induced oncogenesis. Together, inhibition of linc00265 could be a potential strategy for osteosarcoma therapy. It is necessary to mention that *in vivo* mouse study will further validate the function of linc00265 in osteosarcoma development and progression. Moreover, it is required to explore the association between linc00265 levels and prognosis in osteosarcoma patients. In addition, linc00265 could have multiple miRNAs to sponge downstream targets. USP22 and miR-485-5p could have several downstream targets, which need to be further explored in osteosarcoma cells.

## Data Availability Statement

The raw data supporting the conclusions of this article will be made available by the authors, without undue reservation.

## Author Contributions

TC and GS designed this study. TC, JXL, HZ, and JTL performed the experiments and analyzed the data. TC and GS wrote the manuscript. All authors have read and approved the final version of the manuscript.

## Conflict of Interest

The authors declare that the research was conducted in the absence of any commercial or financial relationships that could be construed as a potential conflict of interest.

## Publisher’s Note

All claims expressed in this article are solely those of the authors and do not necessarily represent those of their affiliated organizations, or those of the publisher, the editors and the reviewers. Any product that may be evaluated in this article, or claim that may be made by its manufacturer, is not guaranteed or endorsed by the publisher.
